# Reflecting upon multisource feedback as ‘assessment for learning’

**DOI:** 10.1007/s40037-015-0175-y

**Published:** 2015-03-27

**Authors:** Joan Sargeant

**Affiliations:** Division of Medical Education, Faculty of Medicine, Dalhousie University, Halifax, NS Canada

The paper by Olle ten Cate et al. on ‘User reception of a simple online multisource feedback tool for residents’ in this issue is a welcome segue into considering how best to use multisource feedback (MSF) in education, especially for residents [1]. For a number of years, Ten Cate has maintained an MSF website for Dutch programme directors and their residents, to enable residents to receive formative feedback from medical colleagues, other health care practitioners and patients.

The nationwide offering of MSF is innovative and impressive, and provides an opportunity for reflection from several perspectives. One is seeing MSF through the lens of formative assessment and ‘assessment for learning’, not solely ‘assessment of learning’. Another is consideration of the potential value of adding a self-assessment questionnaire for residents to complete, and the contribution that this might make to the overall impact of the report. A third perspective for consideration is the feedback conversation which occurs between programme directors and their residents about their MSF reports’ and the influence which these conversations may have upon residents’ learning from their reports. Each of these is discussed in more detail below.

As educators we are becoming increasingly aware of our obligation to provide assessment for learning as well as of learning [2]. The notion of assessment for learning appears to fit particularly well with the tenets of competency-based medical education. In competency-based education we think of learners as progressing through various stages or levels from novice through advanced beginner to competence [3], in multiple domains of clinical performance (e.g., the various CanMeds roles) [4]. Assessment with feedback of specific performance in each domain provides the information and guidance which learners require to know how best to progress to the next level or milestone of performance. Multisource feedback is particularly suited to assessing and providing feedback in domains of practice other than the ‘medical expert’; e.g., the communicator, collaborator, professional roles. The MSF performance data can be provided quantitatively using the mean scores of reviewers’ scores, and qualitatively by including narrative reviewer comments. Ten Cate and colleagues report that residents found the narrative comments, offered as ‘tips’ for improvement and ‘tops’ identifying strengths, were much more helpful than the numerical scores. This is important and supports earlier findings about formative feedback; i.e., that narrative comments can provide specific and relevant observations which can inform how and what to improve, while numerical scores can only identify the presence or absence of a performance gap or a need to improve [5]. Seen in this way, MSF which includes narrative comments can prove useful for learning and improvement and in fact, for various reasons as Ten Cate et al. explain, is better suited for formative feedback than summative.

Some models of MSF include a self-assessment questionnaire in addition to questionnaires completed by reviewers. The Dutch model described in this article does not include such a questionnaire and participating programme directors suggested that adding one could be helpful. There are several benefits to adding a specific self-assessment questionnaire comprised of the same items as those completed by reviewers. Research has shown that the presence of the learner’s self-assessment provides additional information as it tells the supervisor how accurately the learner is able to self-assess. If the self-assessment scores are very close to those of external reviewers, they indicate a close match between how learners view their performance and how others do. A gap between the two sets of scores, and especially if the learner rates himself higher than his reviewers, can identify for the supervisor that the learner’s self-assessment may be flawed in some way, and can be the stimulus for a discussion of how the learner arrived at his self-assessment. Self-monitoring and informed self-assessment are professional lifelong responsibilities, and having a tool such as MSF which incorporates self-assessment, can provide insight into a learner’s self-assessment and self-monitoring processes, processes which may otherwise be difficult to uncover [6].

The programme director or supervisor can also play a central role in facilitating the learner’s acceptance and appropriate use of performance data such as provided in MSF, especially in those situations where the learner’s self-assessment ratings differ from those of reviewers. As we know, disconfirming feedback can be difficult for some learners to accept. For others, the way to use the feedback data to improve may be unclear. Engaging learners in reflective discussions of how they self-assessed and rated themselves and why, and of the rationale that their reviewers might have had for rating them as they did, can shed light upon the learner’s views of themselves and of their potential gaps in performance. Such discussions can also enhance the acceptability of their external feedback and point the way to how they might use it for improvement [6] Many factors can get in the way of learners’ using performance feedback, especially MSF, to improve, such as doubting the credibility of the reviewers, being unclear as to what the feedback data mean, or not knowing how to improve. These factors may be more influential in the nonmedical expert domains such as the professional and communicator roles where standards of performance and measures can be less clear. Hence the goal of having a feedback conversation with learners about their MSF results is to maximize their use of the feedback for improvement, by discovering and addressing the factors which might prevent this. For learners receiving relatively high MSF ratings and comments, the feedback conversation can still help them explore an area that they would like to improve and build upon. It can be helpful for supervisors and programme directors to consider their role as that of a coach to guide learners in identifying opportunities for improving and excelling. A coach helps learners explore and use performance feedback data to become the best that they can be [7].

In summary, MSF and the feedback data it provides to learners can truly become an opportunity for ‘assessment for learning’. To ensure that learning occurs, programme directors and supervisors play important facilitation roles. These include ensuring that narrative as well as numerical performance data are provided, enabling learners to inform their self-assessments by completing a self-assessment and comparing it with the external assessment, having a feedback conversation about factors which might influence use of the data, and coaching for learning and improvement.
